# Effects of toll like receptor 4 (TLR4) and toll like receptor 2 (TLR 2) gene polymorphisms on clinical outcomes in acute non-biliary pancreatitis patients

**DOI:** 10.3389/fimmu.2024.1427187

**Published:** 2024-08-16

**Authors:** Ender Anılır, Filiz Özen, İbrahim Halil Yıldırım

**Affiliations:** ^1^ General Surgery, Biruni University Hospital Organ Transplantation and Hepatobiliary Surgery Center, İstanbul, Türkiye; ^2^ Medical Genetics, Istanbul Medeniyet University Göztepe Training and Research Hospital Genetic Department, Istanbul, Türkiye; ^3^ Department of Genetics, Faculty of Veterinary Medicine, Dicle University, Diyarbakır, Türkiye

**Keywords:** toll like receptor, TRL 4, TLR 2, non-biliary pancreatitis, acute pancreatitis

## Abstract

**Introduction:**

In this retrospective study, it was aimed to evaluate effects of Toll Like Receptor 4 (TLR4) and Toll Like Receptor 2 (TLR 2) gene polymorphisms on clinical outcomes in acute non-biliary pancreatitis patients.

**Methods:**

A total of 108 acute non-biliary pancreatitis patients (ANBP) were retrospectively subjected to the study. Gender, age, number of attacks, hospitalization duration, amylase, lipase, aspartate aminotransferase (AST), alanine aminotransferase (ALT), leukocyte, C-reactive protein (CRP), total bilirubin, direct bilirubin, Atlanta score, ultrasonography (USG), Computer Tomography (CT) and patient outcome differences between TLR 4 Rs4986790, TLR 4 Rs4986791 and TLR 2 groups were evaluated.

**Results:**

According to TLR 4 Rs4986790 groups, females were significantly common in AA sequence (AA) group with statistically significant difference (p<0.05). Leukocyte mean of AG sequence (AG) group was significantly higher than of AA group (p<0.05). All parameter differences between TLR 4 Rs4986791 and TLR 2 groups were statistically insignificant (p>0.05). there was a statistically significant correlation between TLR 4 Rs4986790 and gender (r=0.265; p<0.01), Leukocyte (r=0.200; p<0.05) and Pseudocyst (r=0.203; p<0.05). TLR 4 Rs4986790 gene polymorphism had significant effect on leukocyte level in acute non-biliary pancreatitis patients (OR: -0.1.900; p<0.05). Predictive value of leukocyte for TLR 4 Rs4986790 was statistically significant (Area Under Curve: 0.624; p<0.05). For 7.65 leukocyte cut off value, sensitivity for AA gene polymorphism was 84.2% and specificity was 40.5%

**Conclusion:**

Although the clinical and outcome parameters of ANBP patients in terms of TLR 4 Rs4986791 and TLR 2 do not show significant differences, research findings point to the diagnostic value of patients’ leukocyte parameters in determining TLR-4 Rs4986790 ploimorphism groups.

## 1 Introduction

Acute pancreatitis (AP) is an inflammatory disease of the exocrine pancreas that produces excruciating abdominal pain and numerous organ dysfunctions that can progress to pancreatic necrosis and chronic organ failure (1). Worldwide, the prevalence of AP has risen to about 34 instances per 100,000 people each year (2). Treatment consists of supportive care (e.g., fluid resuscitation, assessment of organ function, pain management), provision of interventional treatments (e.g., cholecystectomy, endoscopic sphincterotomy, necrosectomy in case of necrotizing pancreatitis), and provision of adequate nutrition (3). Alcohol consumption and gallstone blockage in the common bile duct are the most frequent causes of AP. Pancreatic divisum, hypercalcemia, idiocentric medication reactions, hypertriglyceridemia, trauma, infections, heredity, abdominal trauma, ischemia, autoimmune, and idiopathy are further categories of causative variables (4). Differentiating between biliary and non-biliary acute pancreatitis can be achieved by the use of imaging techniques, serum aspartate aminotransferase (AST), total bilirubin, and alkaline phosphatase (ALP) values (5).

As members of the larger family of pattern recognition receptors, toll-like receptors (TLRs) are type 1 transmembrane proteins that recognize a wide range of molecular signals, including those linked to endogenous damage and pathogens, damage-associated molecular patterns, and pathogen-associated molecular patterns (6). The pattern-recognizing receptor known as toll-like receptor 4 (TLR4) is expressed by acute myeloid leukemia (AML) cells, many bone marrow stromal cells, and nonleukemic cells involved in inflammation. TLR4 can bind both endogenous and exogenous ligands (7). Depending on the use of the unique adaptor molecules toll-interleukin receptor-domain-containing adapter-inducing interferon-β (TRIF) and myeloid differentiation primary response gene 88 (MyD88), TLR4 signaling is roughly split into two separate pathways (8). With TLR1, TLR6, and occasionally TLR4, TLR2 is the only TLR that can create functional heterodimers with more than two other TLR types. TLR2 also interacts with several molecules that are not TLRs (9). A variety of TLRs plays a significant role in a number of signal transduction pathways, the most important of which are those that control body temperature, energy balance, and cardiac function (10).

It has been demonstrated that TLR4 signaling contributes significantly to the inflammation of acinar cells in acute pancreatitis. Within 24 hours of the beginning of acute pancreatitis, monocytes produce more TLR4, which decreases to normal levels after a week (11). TLR4 has the ability to mediate the activation of nuclear factor (NF)-κB through the myeloid differentiation primary response gene 88 (MyD88)-dependent pathway. This, in turn, induces the expression and release of inflammatory cytokines, such as interleukin (IL)-6 and tumor necrosis factor (TNF)-α, creating a vicious cycle of positive feedback of inflammation (12). In pancreatitis, stimulation-activated TLR2 and TLR4 can lead to an overabundance of cytokine synthesis and release (13). Targeting TLR4, which identifies many damage-associated molecular patterns linked to AP, appears to be the primary option. Although TLR2 has also been connected to AP, very few research have focused solely on this relationship (14). Although there are limited studies on AP with TLR 4 and TLR 2, there are no sufficient studies on the clinical and outcomes of patients with TLR 4 and TLR 2 in non-biliary AP patients. In this research, it was aimed to evaluate effects of Toll Like Receptor 4 (TLR4) and Toll Like Receptor 2 (TLR 2) gene polymorphisms on clinical outcomes in acute non-biliary pancreatitis patients.

## 2 Methods

The study was conducted retrospectively between January 2014 and January 2015 at Hospital. In Türkiye, public hospitals were merged into the Public Hospitals Union in 2011, and in 2017, they were closed again and reconnected to the central administration of public hospitals. For this reason, there were serious problems in the data management and compilation process in hospitals. For this reason, the researchers chose the most reliable and regular data collection date range of the institution they worked in between 2014 and 2015. The analyses related to the gene in question in the study were also an application financed by the hospital in question at that time, and were not continued afterwards. The research is also a pioneering study of the ongoing current investigation and longitudinal comparison of the same parameters. 108 patients diagnosed with ANBP as a result of abdominal with back pain, with higher amylase and lipase values higher than 3 times the normal interval, no image of gallstones in ultrasonography (US), and pancreatic findings in computed tomography (CT) were included in the study retrospectively after obtaining genetic consent for participation (15). Patients were examined as mild moderate and severe ANBP, using the Atlanta Classification to evaluate the differences in genetic polymorphism (16). The ANBP patient group with TLR 4 Rs 4986790 (Asp299Gly) and TLR 4 Rs 4986791 (Thr399Ile) and TLR 2 intron 2 microsatellite polymorphisms was checked against the control group. Patients in the polymorphism groups were also statistically compared according to their demographic data such as age and sex, and indicators of the disease severity such as the number of attacks, Atlanta criteria, and prerenal fascia thickness on CT. Inflammation markers such as CRP (mg/L) and WBC (10^3^/ml) and amylase (U/L) and lipase (U/L) values in the blood were compared statistically between the polymorphism groups. İdiopathic factors, alcohol use, drugs, and autoimmune disease, which were considered as etiology, were statistically evaluated in terms of polymorphism. The patients were informed about the study and their consent forms were obtained. The ethical standards of the respective committees on human experimentation (institutional and national) and with the Helsinki Declaration of 1964 and its later versions were provided. This study was approved by the University Human Experiments Ethics Committee with the decision number 2014-064 and 2014-065.

### 2.1 DNA isolation and examination of genetic polymorphism

In the study, blood samples were collected from 120 patients to investigate genetic polymorphisms of TLR2 and TLR4 genes. However, due to kit shortages in the laboratory, hemolysis and missing clinical data, genetic polymorphisms for TLR4 Rs 4986790 (Asp299Gly) and TLR4 Rs 4986791 (Thr399Ile) could be examined in 108 patients, and TLR2 microsatellite intron 2 gene polymorphism could be examined in 93 patients. Blood samples taken in EDTA tubes from the patient and control groups were stored at -20°C. Then, DNA isolation was performed from with the commercial kit, Bio Basic BS684-250 (Ontario Canada), according to the protocol suggested by the manufacturer. For the TRs0 polymorphism, the following primers were purchased for PCR: F: 5’- ATT-AGC-ATA-CTT-AGA-CTA-CTA-CCT-CCA, TG R: 5´-GAT-CAA-CTT-CTG-AAA AAG-CAT-TCC-CAC-3’ (Determination of the TLR4 Genotype Using Allele-Specific PCR *BioTechniques 31::-: (July 2001)*. The resulting 249 bp amplicon was cut with Nco1 restriction enzyme and run on a 3% agarose gel. On the agarose gel, the heterozygous AG allele was expected to give 249 + 223 + 26 bp bands whereas the homozygous GG allele was expected to give 223 + 26 bp bands. For the TRs1 polymorphism, the following primers were purchased for PCR F: 5´-GGT-TGC-TGT-TCT-CAA AGT-GAT-TTT-GGG-AGA A-3’ and R: 5´- ACC-TGA-AGA-CTG-GAG-AGT-GAG-TTA-AAT GCT-3’ (Determination of the TLR4 Genotype Using Allele-Specific PCR *BioTechniques 31::-: (July 2001)*</i> and the PCR reaction was set. The obtained PCR products (407 bp) were cut with Hinf I (Thermo Scientific) restriction enzyme and the cut product was run on a 3% agarose gel. The heterozygous CT allele was expected to give 407 + 378 + 29 bp bands and the homozygous TT allele was expected to give 378 + 29 bp bands. Gel images with the resulting bands were photographed and analyzed. PCR was performed with primers involving this region, F: 5’-GCA TTG-CTG-AAT-GTA-TCA-GGG A and R: 5’-CTT-GAG-AAA-TGT-TTT-CTA-GGC, to reveal the GT repeats in the TLR2 gene intronic region. PCR products were run in gel electrophoresis with 10% polyacrylamide and the results were imaged and analyzed. Of these repeats, those with 16 or fewer were considered the S allele; those between 17-22 as the M allele; and those greater than 22 as the L allele.

### 2.2 Statistical methods

Nominal and ordinal parameter descriptions were given by frequency analysis, whereas scale parameters were described by means, standard deviations, median and ranges. Kolmogorov Smirnov test was used for normality test of scale variables. Since distributions were non-normal, Mann Whitney U and Kruskal Wallis tests were used. Chi-Square, Fischer’s Exact and Likelihood ratios were used for differences between nominal and ordinal parameters. Spearman’s rho correlation analysis and Generalized Linear Model (Logit) were used for relationship analysis due to linearization deviations (17, 18). SPSS 25.0 for windows was used for analysis at 95% Confidence Interval and 0.05 significance level.

## 3 Results

According to TLR 4 Rs4986790 groups, females were significantly common in AA group with statistically significant difference (p<0.05). Leukocyte mean of AG group was significantly higher than of AA group (p<0.05). Age, number of attacks, hospitalization duration, amylase, lipase, AST, ALT, CRP, total bilirubin, direct bilirubin, Atlanta score, USG, CT and patient outcome differences between TLR 4 Rs4986790 groups were statistically insignificant (p>0.05) (**Table 1**).

**Table 1:** Baseline characteristics, clinical outcomes and difference analysis results between TRL4 Rs4986790 groups.

	TLR4-Rs4986790		p value
	AA (n=74)	AG (n=34)	
Females, n (%)	**45 (60.8)**	11 (32.4)	**0.005**^**a**^
Age, mean ± SD Median (Min-Max)	57.84 ± 12.07 57.00 (36.00-88.00)	56.91 ± 12.88 54.00 (32.00-85.00)	0.585^b^
Attacks, mean ± SD Median (Min-Max)	1.43 ± 1.17 1.00 (1.00-8.00)	1.41 ± 1.33 1.00 (1.00-8.00)	0.394^b^
Hospitalization duration, mean ± SD Median (Min-Max)	5.85 ± 4.94 4.00 (1.00-30.00)	7.03 ± 5.29 6.50 (1.00-28.00)	0.135^b^
Amylase, mean ± SD Median (Min-Max)	494.09 ± 516.13 346.00 (49.00-3821.00)	578.38 ± 500.20 528.00 (60.00-2224.00)	0.311^b^
Lypase, mean ± SD Median (Min-Max)	335.86 ± 457.61 130.50 (8.00-3020.00)	364.56 ± 690.05 120.50 (11.00-3723.00)	0.431^b^
AST, mean ± SD Median (Min-Max)	75.12 ± 136.89 25.00 (12.00-717.00)	39.82 ± 58.55 22.50 (8.00-334.00)	0.305^b^
ALT, mean ± SD Median (Min-Max)	76.78 ± 123.46 22.50 (5.00-598.00)	45.65 ± 57.26 23.00 (5.00-236.00)	0.776^b^
Leukocyte, mean ± SD Median (Min-Max)	9.11 ± 3.26 8.70 (4.00-19.00)	**10.73 ± 4.08** **9.75 (3.60-20.50)**	**0.039**^**b**^
CRP, mean ± SD Median (Min-Max)	9.27 ± 7.58 7.00 (2.00-42.00)	9.94 ± 9.60 8.00 (2.00-47.00)	0.712^b^
Total bilirubin, mean ± SD Median (Min-Max)	1.24 ± 0.91 0.93 (0.29-4.56)	1.05 ± 0.57 0.86 (0.32-2.91)	0.556^b^
Direct bilirubin, mean ± SD Median (Min-Max)	0.34 ± 0.36 0.22 (0.06-2.10)	0.25 ± 0.20 0.17 (0.05-0.97)	0.208^b^
Atlanta Score, n (%)			
Mild	63 (85.1)	30 (88.2)	0.463^c^
Moderate	9 (12.2)	4 (11.8)	
Severe	2 (2.7)	–	
USG Choledocolithiasis, n (%)	2 (2.7)	1 (2.9)	0.682^a^
USG pancreatitis, n (%)	2 (2.7)	2 (5.9)	0.374^a^
USG Inner abdomen fluid, n (%)	7 (9.5)	4 (11.8)	0.476^a^
CT Normal pancreas, n (%)	16 (21.6)	7 (20.6)	0.559^a^
Pancreas expansion, n (%)	34 (45.9)	19 (55.9)	0.226^a^
Pancreatic/Peripancreatic inflammation, n (%)	6 (8.1)	4 (11.8)	0.388^a^
Fluid at one site, n (%)	2 (2.7)	2 (5.9)	0.374^a^
Fluid at more sites, n (%)	4 (5.4)	2 (5.9)	0.617^a^
Edematous, (%)	5 (6.8)	1 (2.9)	0.383^a^
Necrosizing, n (%)	–	1 (2.9)	0.315^a^
Pseudocyst, n (%)	–	2 (5.9)	0.097^a^
Alcohol, n (%)	2 (2.7)	–	0.467^a^
Autoimmune, n (%)	1 (1.4)	1 (2.9)	0.533^a^

a Fisher’s Exact Test.

b Mann Whitney U Test.

c Chi-Square Likelihood Ratio, SD, Standard Deviation.

Bold parameters mean higher value than other group.

Gender, age, number of attacks, hospitalization duration, amylase, lipase, AST, ALT, leukocyte, CRP, total bilirubin, direct bilirubin, Atlanta score, USG, CT and patient outcome differences between TLR 4 Rs4986791 groups were statistically insignificant (p>0.05) (**Table 2**).

**Table 2:** Baseline characteristics, clinical outcomes and difference analysis results between TRL4 RS4986791 groups.

	TLR4 Rs4986791		p value
	CC (n=68)	CT (n=40)	
Females, n (%)	39 (57.4)	17 (42.5)	0.098^a^
Age, mean ± SD Median (Min-Max)	58.43 ± 11.81 58.00 (36.00-88.00)	56.05 ± 13.04 54.00 (32.00-85.00)	0.211^b^
Attacks, mean ± SD Median (Min-Max)	1.46 ± 1.21 1.00 (1.00-8.00)	1.38 ± 1.23 1.00 (1.00-8.00)	0.335^b^
Hospitalization duration, mean ± SD Median (Min-Max)	5.94 ± 5.00 4.00 (1.00-30.00)	6.70 ± 5.18 5.50 (1.00-28.00)	0.315^b^
Amylase, mean ± SD Median (Min-Max)	461.51 ± 343.33 346.00 (65.00-1580.00)	621.13 ± 704.01 522.00 (49.00-3821.00)	0.488^b^
Lypase, mean ± SD Median (Min-Max)	338.31 ± 454.65 143.00 (8.00-3020.00)	356.10 ± 663.36 107.50 (11.00-3723.00)	0.319^b^
AST, mean ± SD Median (Min-Max)	75.74 ± 137.91 28.50 (12.00-717.00)	44.08 ± 73.47 23.00 (8.00-351.00)	0.176^b^
ALT, mean ± SD Median (Min-Max)	79.85 ± 128.18 21.50 (5.00-598.00)	45.10 ± 53.67 23.00 (5.00-236.00)	0.949^b^
Leukocyte, mean ± SD Median (Min-Max)	9.10 ± 3.26 8.70 (4.00-19.00)	10.50 ± 4.02 9.55 (3.60-20.50)	0.054^b^
CRP, mean ± SD Median (Min-Max)	9.40 ± 7.79 7.50 (3.00-42.00)	9.63 ± 9.02 7.50 (2.00-47.00)	0.630^b^
Total bilirubin, mean ± SD Median (Min-Max)	1.25 ± 0.93 0.91 (0.29-4.56)	1.06 ± 0.58 0.87 (0.32-2.91)	0.681^b^
Direct bilirubin, mean ± SD Median (Min-Max)	0.36 ± 0.37 0.23 (0.06-2.10)	0.24 ± 0.19 0.16 (0.05-0.97)	0.092^b^
Atlanta Score, n (%)			
Mild	58 (85.3)	35 (87.5)	0.391^c^
Moderate	8 (11.8)	5 (12.5)	
Severe	2 (2.9)	–	
USG Choledocolithiasis, n (%)	2 (2.9)	1 (2.5)	0.692^a^
USG pancreatitis, n (%)	2 (2.9)	2 (5.0)	0.474^a^
USG Inner abdomen fluid, n (%)	7 (10.3)	4 (10.0)	0.618^a^
CT Normal pancreas, n (%)	13 (19.1)	10 (25.0)	0.313^a^
Pancreas expansion, n (%)	31 (45.6)	22 (55.0)	0.228^a^
Pancreatic/Peripancreatic inflammation, n (%)	6 (8.8)	4 (10.0)	0.545^a^
Fluid at one site, n (%)	2 (2.9)	2 (5.0)	0.474^a^
Fluid at more sites, n (%)	4 (5.9)	2 (5.0)	0.607^a^
Edematous, (%)	5 (7.4)	1 (2.5)	0.275^a^
Necrosizing, n (%)	–	1 (2.5)	0.370^a^
Pseudocyst, n (%)	–	2 (5.0)	0.135^a^
Alcohol, n (%)	2 (2.9)	–	0.394^a^
Autoimmune, n (%)	1 (1.5)	1 (2.5)	0.606^a^

a Fisher’s Exact Test.

b Mann Whitney U Test.

c Chi-Square Likelihood Ratio, SD, Standard Deviation.

According to difference analysis results; gender, age, number of attacks, hospitalization duration, amylase, lipase, AST, ALT, leukocyte, CRP, total bilirubin, direct bilirubin, Atlanta score, USG, CT and patient outcome differences between TLR 2 groups were statistically insignificant (p>0.05) (**Table 3**).

**Table 3:** Baseline characteristics, clinical outcomes and difference analysis results between TRL2 groups.

	TLR2			p value
	ML (n=58)	LL (n=4)	MS (n=31)	
Females, n (%)	28 (48.3)	4 (100.0)	17 (54.8)	0.060^a^
Age, mean ± SD Median (Min-Max)	58.02 ± 12.52 56.00 (32.00-85.00)	64.50 ± 14.62 59.50 (54.00-85.00)	54.32 ± 11.49 53.00 (36.00-88.00)	0.219^b^
Attacks, mean ± SD Median (Min-Max)	1.52 ± 1.38 1.00 (1.00-8.00)	1.50 ± 0.58 1.50 (1.00-2.00)	1.39 ± 1.23 1.00 (1.00-7.00)	0.271^b^
Hospitalization duration, mean ± SD Median (Min-Max)	6.45 ± 5.84 4.00 (1.00-30.00)	8.25 ± 4.57 8.50 (3.00-13.00)	5.35 ± 3.90 4.00 (1.00-20.00)	0.421^b^
Amylase, mean ± SD Median (Min-Max)	497.09 ± 404.05 454.50 (49.00-2224.00)	268.50 ± 225.44 219.50 (81.00-554.00)	535.65 ± 418.12 452.00 (65.00-2124.00)	0.287^b^
Lypase, mean ± SD Median (Min-Max)	368.55 ± 565.09 158.50 (15.00-3723.00)	180.00 ± 255.14 68.00 (23.00-561.00)	328.03 ± 545.57 134.00 (11.00-3020.00)	0.526^b^
AST, mean ± SD Median (Min-Max)	52.60 ± 93.54 23.00 (8.00-599.00)	33.00 ± 14.49 28.00 (22.00-54.00)	78.45 ± 140.03 25.00 (12.00-717.00)	0.625^b^
ALT, mean ± SD Median (Min-Max)	79.34 ± 120.13 23.00 (5.00-598.00)	52.25 ± 35.28 54.00 (14.0087.00)	60.65 ± 110.17 19.00 (5.00-501.00)	0.363^b^
Leukocyte, mean ± SD Median (Min-Max)	9.58 ± 3.97 8.70 (3.60-20.50)	8.52 ± 4.70 7.60 (4.00-14.90)	9.77 ± 3.45 9.00 (4.85-19.00)	0.681^b^
CRP, mean ± SD Median (Min-Max)	9.57 ± 8.71 7.00 (2.00-47.00)	6.25 ± 2.63 6.00 (4.00-9.00)	9.77 ± 8.61 8.00 (3.00-42.00)	0.732^b^
Total bilirubin, mean ± SD Median (Min-Max)	1.26 ± 0.88 0.96 (0.32-4.56)	0.90 ± 0.18 0.91 (0.72-1.07)	1.09 ± 0.79 0.82 (0.29-4.22)	0.328^b^
Direct bilirubin, mean ± SD Median (Min-Max)	0.30 ± 0.30 0.20 (0.05-1.52)	0.22 ± 0.08 0.24 (0.11-0.29)	0.35 ± 0.39 0.23 (0.08-2.10)	0.689^b^
Atlanta Score, n (%)				
Mild	49 (84.5)	3 (75.0)	29 (93.5)	0.492^a^
Moderate	7 (12.1)	1 (25.0)	2 (6.5)	
Severe	2 (3.4)	–	–	
USG Choledocolithiasis, n (%)	1 (1.7)	–	1 (3.2)	0.829^a^
USG pancreatitis, n (%)	3 (5.2)	–	1 (4.3)	0.761^a^
USG Inner abdomen fluid, n (%)	6 (10.3)	–	3 (9.7)	0.656^a^
CT Normal pancreas, n (%)	13 (22.4)	1 (25.0)	8 (25.8)	0.936^a^
Pancreas expansion, n (%)	26 (44.8)	3 (75.0)	12 (38.7)	0.376^a^
Pancreatic/Peripancreatic inflammation, n (%)	6 (10.3)	–	2 (6.5)	0.569^a^
Fluid at one site, n (%)	1 (1.7)	–	2 (6.5)	0.456^a^
Fluid at more sites, n (%)	5 (8.6)	–	–	0.087^a^
Edematous, (%)	4 (6.9)	–	–	0.143^a^
Necrosizing, n (%)	1 (1.7)	–	–	0.622^a^
Pseudocyst, n (%)	2 (3.4)	–	–	0.384^a^
Alcohol, n (%)	1 (1.7)	–	1 (3.2)	0.829^a^
Autoimmune, n (%)	1 (1.7)	–	1 (3.2)	0.829^a^

a Chi-Square Likelihood Ratio.

b Kruskal Wallis Test, SD, Standard Deviation.

Spearman’s rho correlation analysis results showed that there was a statistically significant correlation between TLR 4 Rs4986790 and gender (r=0.265; p<0.01), Leukocyte (r=0.200; p<0.05) and Pseudocyst (r=0.203; p<0.05) (**Table 4**). However, correlations between patient outcomes and TRL 4 Rs4986791 and TLR 2 were statistically insignificant (p>0.05) (**Table 4**).

**Table 4:** Spearman’s rho correlation analysis results between clinical parameters, TLR 4 Rs4986790, TLR 4 Rs4986791 and TLR 2 groups.

	TLR4 Rs4986790	TLR4 Rs4986791	TLR2
Gender	0.265^**^	0.144	-0.086
Age	-0.053	-0.121	-0.115
Attacks	-0.082	-0.093	-0.098
Hospitalization duration	0.144	0.097	-0.037
Amylase	0.098	0.067	0.051
Lypase	-0.076	-0.096	-0.047
AST	-0.099	-0.131	0.086
ALT	-0.028	0.006	-0.120
Leukocyte	0.200^*^	0.186	0.055
CRP	-0.036	-0.047	0.018
Total bilirubin	-0.057	-0.040	-0.153
Direct bilirubin	-0.122	-0.163	0.090
Atlanta score	-0.046	-0.036	-0.119
Baltazar score	0.128	0.094	-0.096
USG Choledecolithiasis	0.007	-0.013	0.044
USG Pancreatitis	0.078	0.053	-0.050
USG Inner abdomen fluid	0.035	-0.005	-0.019
CT normal pancreas	-0.012	0.069	0.038
Pancreas xpansion	0.092	0.091	-0.040
Pancreatic Peripancreatic inflammation	0.059	0.020	-0.072
Liquid at one site	0.078	0.053	0.119
Liquid at more site	0.010	-0.019	-0.183
Edematous	-0.077	-0.102	-0.163
Necrosizing	0.143	0.126	-0.080
Pseudocyst	0.203^*^	0.179	-0.114
Alcohol	-0.093	-0.105	0.044
Autoimmune	0.055	0.037	0.044

*p<0.05, **p<0.01.

Generalized Linear Model (Logit) analysis results showed that TLR 4 Rs4986790 gene polymorphism had significant effect on leukocyte level in acute non-biliary pancreatitis patients (OR: -0.1.900; p<0.05) (**Table 5**).

**Table 5:** Generalized Linear Model (Logit) for effect of TLR 4 Rs4986790 on Leukocyte.

Parameter	B	Std. Error	95% Wald Confidence Interval		Hypothesis Test		
			Lower	Upper	Wald Chi-Square	df	p
(Intercept)	6.873	2.4751	2.022	11.724	7.712	1	0.005
[Gender=Female]	.153	.6930	-1.205	1.512	.049	1	.825
[Gender=Male]	0^a^	.	.	.	.	.	.
[Pseudocyst=No]	4.042	2.5295	-.916	8.999	2.553	1	0.110
[Pseudocyst=Yes]	0^a^	.	.	.	.	.	.
[TLR4_Rs4986790=AA]	-1.900	.7614	-3.392	-.407	6.226	1	0.013
[TLR4_Rs4986790=AG]	0^a^	.	.	.	.	.	.
(Scale)	12.012^b^	1.6346	9.200	15.684			

Dependent Variable: Leukocyte, Model: (Intercept), Gender, Pseudocyst, TLR4_Rs4986790.

a Set to zero because this parameter is redundant.

b Maximum likelihood estimate.

**Figure 1:**
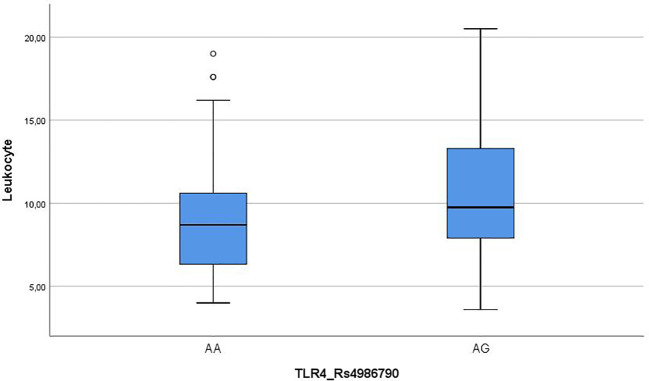
Leukocyte means and rage differences between TLR 4 Rs4986790 groups.

Leukocyte mean of AA group was significantly lower than AG group, and leukocyte change range was also lower in the AA group (**Figure 1**).

**Figure 2:**
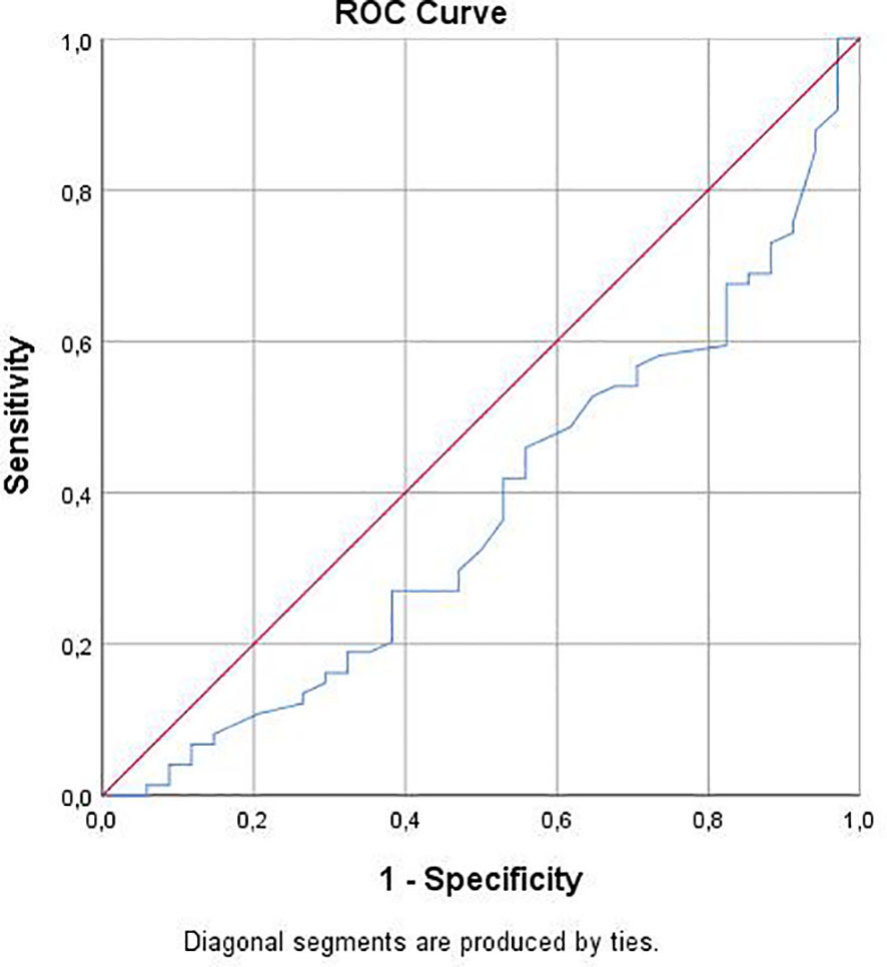
ROC Curve for leukocyte and TLR 4 Rs4986790.

ROC analysis results showed that predictive value of leukocyte for TLR 4 Rs4986790 was statistically significant (Area Under Curve: 0.624; p<0.05). For 7.65 leukocyte cut off value, sensitivity for AA gene polymorphism was 84.2% and specificity was 40.5% (**Figure 2**).

## 4 Discussion

In this study, we aimed to examine the effects of TLR 2 and TLR 4 polymorphism levels on the clinical and outcomes of patients in ANBP patients, and in this context, through the files of 108 patients, the effect of two members of the TLR group on ANBP, which are most associated with AP, but not enough studies have been conducted on non-biliary AP. was evaluated.

Studies on TLR 4 in AP patients are limited, and in general, in these studies, TLR 4 has been associated with overabundance of cytokine synthesis and release (19–21). In our study, only gender and leukocyte showed statistically significant differences between Rs4986790 groups of TLR 4. Age, number of attacks, hospitalization duration, amylase, lipase, AST, ALT, CRP, total bilirubin, direct bilirubin, Atlanta score, USG, CT and patient outcome differences between TLR 4 Rs4986790 groups were statistically insignificant. Although limited in the literature, studies on AP have reported that TLR 4 may be associated with both the clinical features and prognosis of the disease and its outcomes. However, in our study, the fact that it was significant only for one type of TLR 4 and only for one parameter indicates that it would be useful to focus more on the difference between AP and ANBP.

Similar to TLR 4, there are studies associating TLR 2 with clinical and outcomes, especially inflammation, in AP patients. Although the sample and data are limited in these studies, further studies are pointed out for the use of TLR 2 from the TLR family as a clinical prognosis in AP patients (22–25). In our study, clinical values ​​and patient outcomes did not show a statistically significant difference in both TLR 2 groups in ANBP patients. Although there may be many reasons for this, it is possible to state that when ANBP is included, disease prognoses do not change according to TLR 2 groups and this has an effect. In addition, it is possible to say that the fact that the study was conducted in a single center and the demographic and clinical characteristics of the patients were close to each other had an impact. It is possible to obtain more expanded results in larger sample and multicenter studies.

### 4.1 Limitations of the study

Although the study provides some interesting insight to leukocyte correlation with a specific TLR4 polymorphism, it has limitations in terms of the patient population, information on samples, depth and scope of the disease. Although there are significant developments in both literature and medical field regarding gene mutation, gene determination and polymorphism, these technologies are limited in public hospitals and public health units in general due to high cost and low follow-up. In this context, the research can be shown as a source for further studies.

The most important limitation of the research is that clinical applications related to polymorphism are costly, so it is difficult to obtain this data without a specific research period or a specific sponsor. Therefore, there are not enough multicenter studies focusing on a large number of patients. Although the study was taken retrospectively, the data are limited to the date of these analyses.

Another important limitation of the research is that the studies on ANBP are limited and the definition of ANBP extends to a very wide range. Although BP patients are relatively easily diagnosed and their characteristics are defined in AP, this is not the case for ANBP. For this reason, there are not enough studies in the literature to compare the research results. This also brings the research to the fore as a pioneer in the field.

In the relational screening model of the study, correlation analysis results revealed a significant relationship only between TLR 4 Rs4986790 groups and gender, leukocyte and Pseudocyst. There was no statistically significant relationship for TLR 4 Rs4986791 and TLR 2. In the regression analysis (Logit model), only leukocyte had a significant effect among the TLR 4 Rs4986791 groups. It was found by ROC analysis that the leukocyte and TLR 4 Rs4986791 groups had diagnostic value.

### 4.2 Contribution to literature

AP is a pancreatic disease that reduces the quality of life, is associated with many diseases, and is important for public health. However, studies on ANBP within AP are quite limited. However, a good understanding of the ANBP mechanism may make positive contributions to AP-related disease and prognosis factors. In addition, revealing the effect of genetic morphism in ANBP disease may provide an important basis for further genetic studies. In this respect, the research conducted is important in that it focuses on genetic morphism and ANBP.

Another important contribution of the research is that it may be possible to predict clinically the diagnosis and determination of TLR 4 Rs4986790 groups with leukocyte detection. If further studies are conducted between gene mutation and polymorphism and leukocytes and the relationship between them has a strong diagnostic value, clinical predictions can be made based on leukocytes in the future. However, sensitivity and specificity studies are needed. In this regard, this research may be a source for this benefit. Leukocyte infiltration and blood counts are highly variable among individuals with acute pancreatitis across a wide range of etiologies. For this reason, multi centered and variable researches are needed. In further studies, when the importance of the differences between TLR 4 Rs4986790 groups in the disease-related process is revealed in AP patients or other diseases in general, the research results can be considered as a basic study as it allows TLR 4 Rs4986790 group determination to be easy, practical and low-cost.

## 5 Conclusion

The clinical and outcome parameters of ANBP patients in terms of TLR 4 Rs4986791 and TLR 2 do not show significant differences. However, research findings showed that the diagnostic value of patients’ leukocyte parameters in determining TLR-4 Rs4986790 ploimorphism groups was statistically significant. Leukocyte can be used as an effective indicator for ANBP patients in the detection of gene polymorphism and TLR 4 Rs4986791 groups and the clinical use of features for these groups.

## Data Availability

The datasets presented in this article are not readily available because the data is not public in accordance with the ethical approval for this study. Requests to access the datasets should be directed to corresponding author: Ender Anılır, dr.enderanilir@gmail.com.

## References

[1] Szatmary P Grammatikopoulos T Cai W Huang W Mukherjee R Halloran C. Acute pancreatitis: diagnosis and treatment. Drugs. (2022) 82:1251–76. doi: 10.1007/s40265-022-01766-4 PMC9454414 36074322

[2] Zheng Z Ding YX Qu YX Cao F Li F. A narrative review of acute pancreatitis and its diagnosis, pathogenetic mechanism, and management. Ann Transl Med. (2021) 9:69. doi: 10.21037/atm-20-4802 33553362 PMC7859757

[3] Walkowska J Zielinska N Tubbs RS Podgórski M Dłubek-Ruxer J Olewnik Ł. Diagnosis and treatment of acute pancreatitis. Diagnostics (Basel). (2022) 12:1974. doi: 10.3390/diagnostics12081974 36010324 PMC9406704

[4] Anilir E Ozen F Yildirim IH Ozemir IA Ozlu C Alimoglu O. IL-8 gene polymorphism in acute biliary and non biliary pancreatitis: probable cause of high level parameters? Ann Hepatobiliary Pancreat Surg. (2017) 21:30–8. doi: 10.14701/ahbps.2017.21.1.30 PMC5353913 28317043

[5] Okuturlar Y Soylu A Dogan H Cakmak S Kirac Utku I Oztosun B. Mean platelet volume in patients with biliary and non-biliary acute pancreatitis. Int J Clin Exp Pathol. (2015) 8:2051–6. PMC4396319 25973103

[6] Tam JSY Coller JK Hughes PA Prestidge CA Bowen JM. Toll-like receptor 4 (TLR4) antagonists as potential therapeutics for intestinal inflammation. Indian J Gastroenterol. (2021) 40:5–21. doi: 10.1007/s12664-020-01114-y 33666891 PMC7934812

[7] Bruserud Ø Reikvam H Brenner AK. Toll-like receptor 4, osteoblasts and leukemogenesis; the lesson from acute myeloid leukemia. Molecules. (2022) 27:735. doi: 10.3390/molecules27030735 35163998 PMC8838156

[8] Zhang P Yang M Chen C Liu L Wei X Zeng S. Toll-like receptor 4 (TLR4)/opioid receptor pathway crosstalk and impact on opioid analgesia, immune function, and gastrointestinal motility. Front Immunol. (2020) 11:1455. doi: 10.3389/fimmu.2020.01455 32733481 PMC7360813

[9] Di Lorenzo A Bolli E Tarone L Cavallo F Conti L. Toll-like receptor 2 at the crossroad between cancer cells, the immune system, and the microbiota. Int J Mol Sci. (2020) 21:9418. doi: 10.3390/ijms21249418 33321934 PMC7763461

[10] Li MJ Yan SB Dong H Huang ZG Li DM Tang YL. Clinical assessment and molecular mechanism of the upregulation of Toll-like receptor 2 (TLR2) in myocardial infarction. BMC Cardiovasc Disord. (2022) 22:314. doi: 10.1186/s12872-022-02754-y 35840880 PMC9287878

[11] Mattke J Darden CM Lawrence MC Kuncha J Shah YA Kane RR. Toll-like receptor 4 in pancreatic damage and immune infiltration in acute pancreatitis. Front Immunol. (2024) 15:1362727. doi: 10.3389/fimmu.2024.1362727 38585277 PMC10995222

[12] Huang SQ Wen Y Sun HY Deng J Zhang YL Huang QL. Abdominal paracentesis drainage attenuates intestinal inflammation in rats with severe acute pancreatitis by inhibiting the HMGB1-mediated TLR4 signaling pathway. World J Gastroenterol. (2021) 27:815–34. doi: 10.3748/wjg.v27.i9.815 PMC7941863 33727772

[13] Zhang L Wu HS Chen Y Guo XJ Wang L Wang CY. Role of nitric oxide in Toll-like receptor 2 and 4 mRNA expression in liver of acute hemorrhagic necrotizing pancreatitis rats. World J Gastroenterol. (2006) 12:485–8. doi: 10.3748/wjg.v12.i3.485 PMC4066075 16489656

[14] Vaz J Akbarshahi H Andersson R. Controversial role of toll-like receptors in acute pancreatitis. World J Gastroenterol. (2013) 19:616–30. doi: 10.3748/wjg.v19.i5.616 PMC3574587 23431068

[15] Kimura Y Takada T Kawarada Y Hirata K Mayumi T Yoshida M. JPN guidelines for the management of acute pancreatitis: treatment of gallstone-induced acute pancreatitis. J Hepatobiliary Pancreat Surg. (2006) 13:56–60. doi: 10.1007/s00534-005-1052-6 16463212 PMC2779396

[16] Banks PA Bollen TL Dervenis C Gooszen HG Johnson CD Sarr MG. Classification of acute pancreatitis–2012: revision of the atlanta classification and definitions by international consensus. Gut. (2013) 62:102–11. doi: 10.1136/gutjnl-2012-302779 23100216

[17] Yılmaz K Turanlı M. A multi-disciplinary investigation of linearization deviations in different regression models. Asian J Probability Statistics. (2023) 22:15–9. doi: 10.9734/ajpas/2023/v22i3484

[18] Turanlı M Yılmaz K. A Multi-disciplinary Investigation of Linearization Deviations in Different Regression Models. Asian Journal of Medicine and Health (2022).

[19] van den Berg FF Kempeneers MA van Santvoort HC Zwinderman AH Issa Y Boermeester MA. Meta-analysis and field synopsis of genetic variants associated with the risk and severity of acute pancreatitis. BJS Open. (2020) 4:3–15. doi: 10.1002/bjs5.50231 32011822 PMC6996643

[20] Zhou X Jin S Pan J Lin Q Yang S Lu Y. Relationship between cholesterol-related lipids and severe acute pancreatitis: from bench to bedside. J Clin Med. (2023) 12:1729. doi: 10.3390/jcm12051729 36902516 PMC10003000

[21] Vaz J Andersson R. Intervention on toll-like receptors in pancreatic cancer. World J Gastroenterol. (2014) 20:5808–17. doi: 10.3748/wjg.v20.i19.5808 PMC4024790 24914341

[22] Orlacchio A Mazzone P. The role of toll-like receptors (TLRs) mediated inflammation in pancreatic cancer pathophysiology. Int J Mol Sci. (2021) 22:12743. doi: 10.3390/ijms222312743 34884547 PMC8657588

[23] Zhao R Song C Liu L Liu Q Zhou N Zhou X. Single immunoglobulin and Toll-interleukin-1 receptor domain containing molecule protects against severe acute pancreatitis in *vitro* by negatively regulating the Toll-like receptor-4 signaling pathway: A clinical and experimental study. Mol Med Rep. (2020) 22:2851–9. doi: 10.3892/mmr.2020.11379 PMC7453662 32945488

[24] Shen X Li WQ. High-mobility group box 1 protein and its role in severe acute pancreatitis. World J Gastroenterol. (2015) 21:1424–35. doi: 10.3748/wjg.v21.i5.1424 PMC4316085 25663762

[25] Anılır E Özen F Özemir İA Yıldırım İH Bilgiç Ç Alimoğlu O. TLR4 Asp299Gly and Thr399Ile and TLR2 intron 2 microsatellite gene polymorphism in patients with acute biliary pancreatitis: Does it cause the disease? Turk J Surg. (2018) 34:191–7. doi: 10.5152/turkjsurg.2017.3828 PMC6173596 30216179

